# The Enterprise2 Stent for Endovascular Treatment of Intracranial Aneurysms: Short-Term Results From a Single Center Experience

**DOI:** 10.3389/fneur.2020.589689

**Published:** 2020-12-03

**Authors:** Linhui Chen, Chaobo Zheng, Jiong Wu, Jie Gong, Yuhai Gao, Shu Wan

**Affiliations:** Brain Center Department, Zhejiang Hospital, Hangzhou, China

**Keywords:** aneurysm, endovascular treatment, stent, enterprise 2 stent, incomplete stent apposition (ISA)

## Abstract

**Background:** Self-expanding devices, such as the Enterprise VRD (EP-VRD) have widely used for stent-assisted coiling treatment in wided-necked aneuryms while some thromboembolic complications were reported due to its incomplete stent apposition (ISA). We report our experiences on the novel Enterprise2 (EP-VRD2) stent *in vivo* in the treatment of intracranial and cranial cervical junction aneurysms.

**Methods:** Twenty-five consecutive patients with intracranial or cranial cervical junction aneurysms were treated with EP-VRD2 stents retrospectively collected in our institution. We use the ‘jailing' technique in all cases and deployed the stent by using pushing over the outer curve technique. The 3- or 6-monthS follow-up was done regularly by DSA.

**Results:** Twenty-five EP-VRD2 stents were implanted to treat 21 aneurysms at the siphon segment of internal carotid artery (ICA), one at the petrous segment, two at the cervical segment, one at the verteral artery with five accompanied with stenosis. Two patients had kinking during the procedure and were solved by microwire or microcatheter massaging. Four patients with a larger arc angle and a smaller radius of the parent vessel was detected ISA. No patient underwent the ischemic event after the operation. Twenty-three of 25 patients were evaluated after 3- or 6-months by DSA, 22 showed complete occlusion (RROC1), one slight re-stenosis in the follow-up within those five patients with stenosis. A length of 23 mm seemed associated with ISA (*p* < 0.01).

**Conclusion:** The EP-VRD2 performed well in our small patient series; however, ISA could still occur with a sharp angle of the parent vessel.

## Introduction

Currently, novel endovascular devices and technologies have increased opportunities for treatment of intracranial aneurysms (IAs). These include surgical clipping, even in complex aneurysms. Among these, stent-assisted coiling (SAC) has become the modality of choice for treatment of wide-necked aneurysms due to its lower recurrence rates compared to coiling alone (1–4). The Enterprise Vascular Reconstruction Device (EP-VRD, Codman Neurovascular, Raynham, MA, USA) is a self-expandable laser device that is the first closed-cell designed stent. It has been widely used in SAC treatment of IAs (5–7). Nevertheless, there remain some drawbacks limiting its application. Johnson et al. reported that 4.4–6.4% (8) of patients who underwent SAC treatment had thromboembolic complications partially due to the incomplete stent apposition (ISA) in blood vessels with large diameters and curvatures (9).

To overcome this weakness, the Enterprise2 stent (EP-VRD2) was developed; it is the second-generation Enterprise stent, featuring improved design in terms of geometry. EP-VRD2 showed better wall apposition in curved vessels than did EP-VRD in an *in vitro* study (10). Clinicians could further reduce ISA in curved vessels by pushing over the outer curve deploying technique (11). Nevertheless, little is known about its performance *in vivo* except first experiences reported by Herweh et al. (12).

In this study, we enrolled a series of 25 cases of intracranial or cranial cervical junction aneurysms treated with EP-VRD2 as well as its short-term follow-up results. We then preliminarily analyzed its wall apposition performance to confirm its safety and efficacy in a Chinese population.

## Materials and Methods

### Patients

We retrospectively collected the data from 25 patients with intracranial or cranial cervical junction aneurysms treated with EP-VRD2 from November 2018 to December 2019 at our institution. Informed consent for participants was obtained from the patients or their legal representatives, and the protocol was approved by our ethical committee.

Standard dual-antiplatelet therapy (100 mg/d aspirin and 75 mg/d clopidogrel, loading dose 300 mg once each) was administered 5 days prior to the procedure for unruptured cases and continued thereafter. For ruptured aneurysms, patients were loaded with an appropriate weight-based dose of tirofiban (6 μg/kg once, and then 0.1 μg/kg per minute for 12 h) at the moment of stent deployment then changed to loading-dosed dual-antiplatelet drugs for 6–8 h and discontinuation of tirofiban. General anesthesia was used in all patients. We measured aneurysms using digital subtraction angiography (DSA) and 3-dimensional (3D) rotational angiography. After the procedure, patients were given standard dual-antiplatelet therapy for 3 months and only aspirin (100 mg/d) thereafter. The procedure-related complications and immediate and 3–6 monthly follow-up DSA results were reviewed.

### Procedure

All endovascular procedures were performed using Allura Xper FD20 angiographic system (Philips, Netherlands). We used the “jailing” technique in all cases. Either a 6-F guide catheter or a combination of a 6-F-long sheath and a 6-F intermediate catheter was positioned into the carotid or vertebral artery, depending on the distortion of the vascular access. 3D imaging and 2D imaging of the target aneurysms were performed to evaluate the tortuous conditions and measure related aneurysmal morphological parameters. A pre-shaped 0.014-inch 200-cm micro-guidewire (Synchro, Stryker, Kalamazoo, MI, USA) and a 0.021-inch microcatheter (Prowler Select Plus, Codman Neurovascular, Raynham, MA, USA) for stent delivery according to the manufacturer's instructions were positioned at least 10 mm past the distal location of the aneurysm neck. Coil embolization was performed using a 0.017-inch microcatheter (Echelon-10, ev3 Endovascular, Plymouth, MN, USA). The distal markers of the stent were positioned distally across the aneurysm neck. The distal part of stent was unsheathed by slowly withdrawing the microcatheter and carefully pushing or pulling the delivery wire of stent for adequate expansion and neck coverage. After coil embolization, the delivery wire was further advanced to a position along the outer curve of the parent artery, and the stent was then pushed out completely with the delivery wire maintained along the outer curve (pushing over outer curve technique). Each deploying procedure included an angiography and an Xper CT with diluted contrast (1:5) right after complete deployment to confirm its correct stent placement and satisfactory vessel wall apposition.

### Complications and Follow-Up

In our institution, short-term follow-up DSA was routinely performed 3–6 months after treatment. Telephone or outpatient interview was conducted monthly to investigate potential complications. Procedure-related complications included aneurysm rupture and embolic events [such as transient ischemic attack [TIA] or symptomatic ischemic stroke]. The post-procedure potential-related complications were evaluated with the modified Rankin score (mRS). Occlusion rates were evaluated during the immediate post-embolization and follow-up DSA images for every aneurysm with the Raymond–Roy occlusion classification (RROC) (13): I, complete occlusion; II, neck remnant; and III, residual sac. Any further contrast remnant of the neck or sac during follow-up was defined as “recurrence”.

### Statistical Analysis

Continuous data were presented as mean ± SD. A comparative analysis between the complete and incomplete stent apposition groups was performed using an independent-samples *t*-test. Statistical significance was defined as *P* < 0.05. Statistical analysis was carried out with SPSS Statistics for Windows, Version 26.0 (SPSS Inc., Chicago, Illinois, USA).

## Results

**Figure 1:**
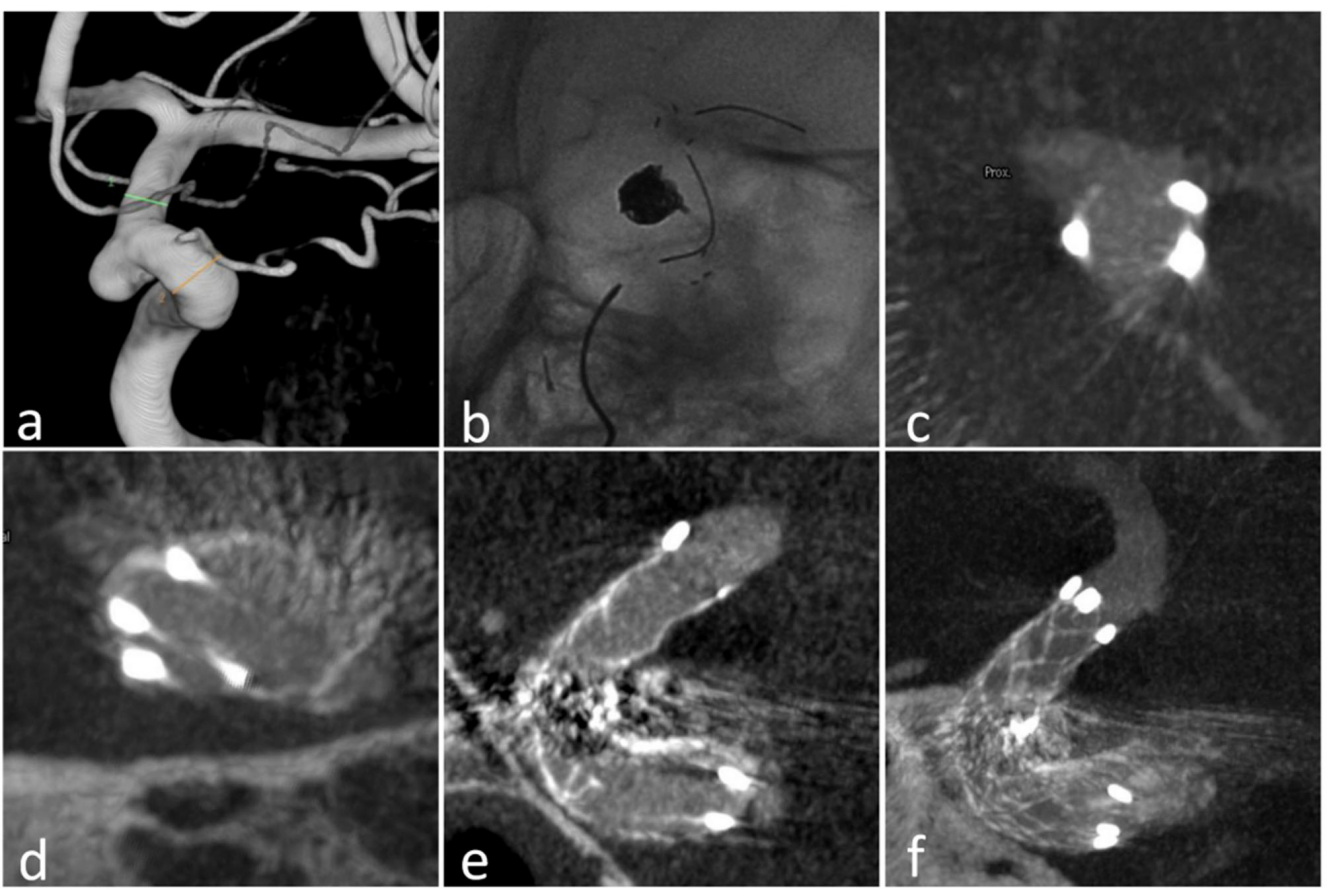
Case 18: **(a)** the aneurysm at the ophthalmic arterial segment of ICA; **(b)** the stent (23 mm) in the ICA; **(c)** the proximal markers were asymmetrical; **(d)** the distal markers were symmetrical; **(e)** sagittal view of the stent in Xper CT; **(f)** correct stent wall position of the deployed stent with larger thickness in Xper CT.

**Figure 2:**
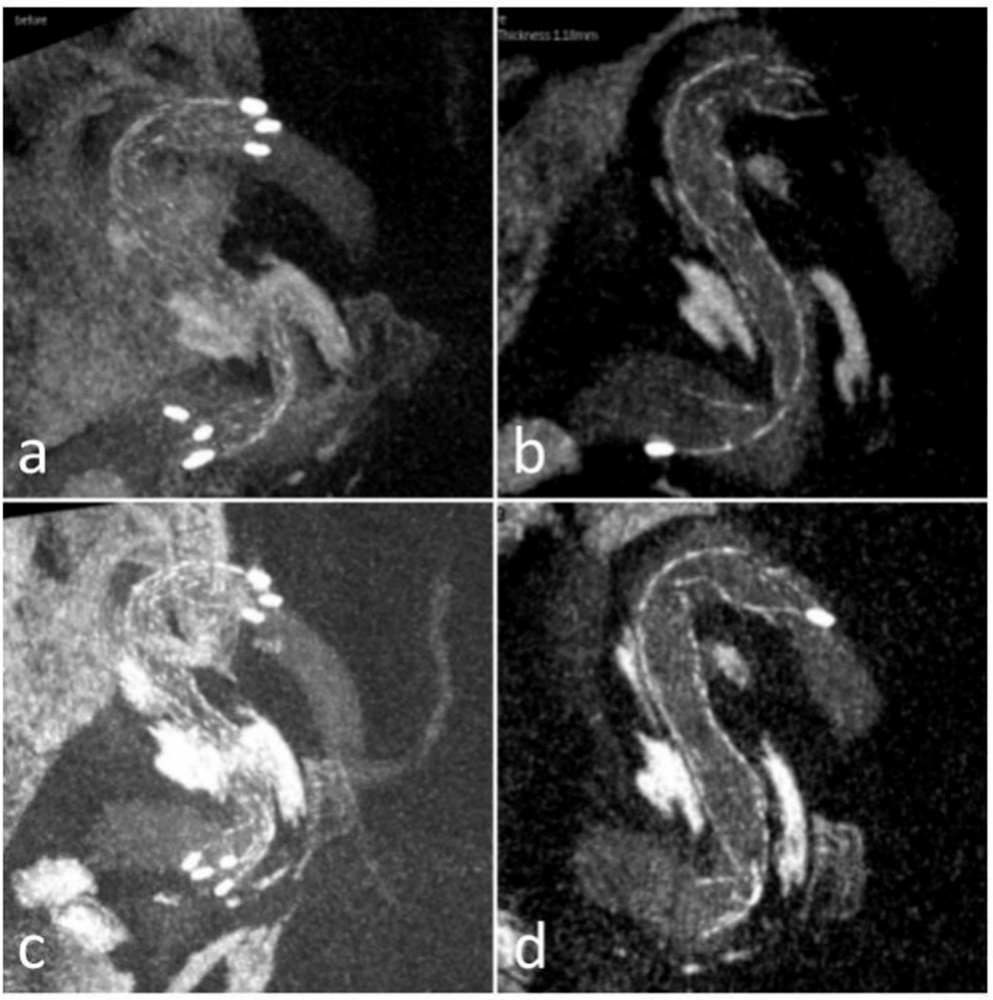
Case 23 **(a,b)** sagittal views of the stent in Xper CT during the procedure showed the kinking; **(c,d)** sagittal views of the stent after massaging via microcatheter or microwire showed the kinking improved.

**Figure 3:**
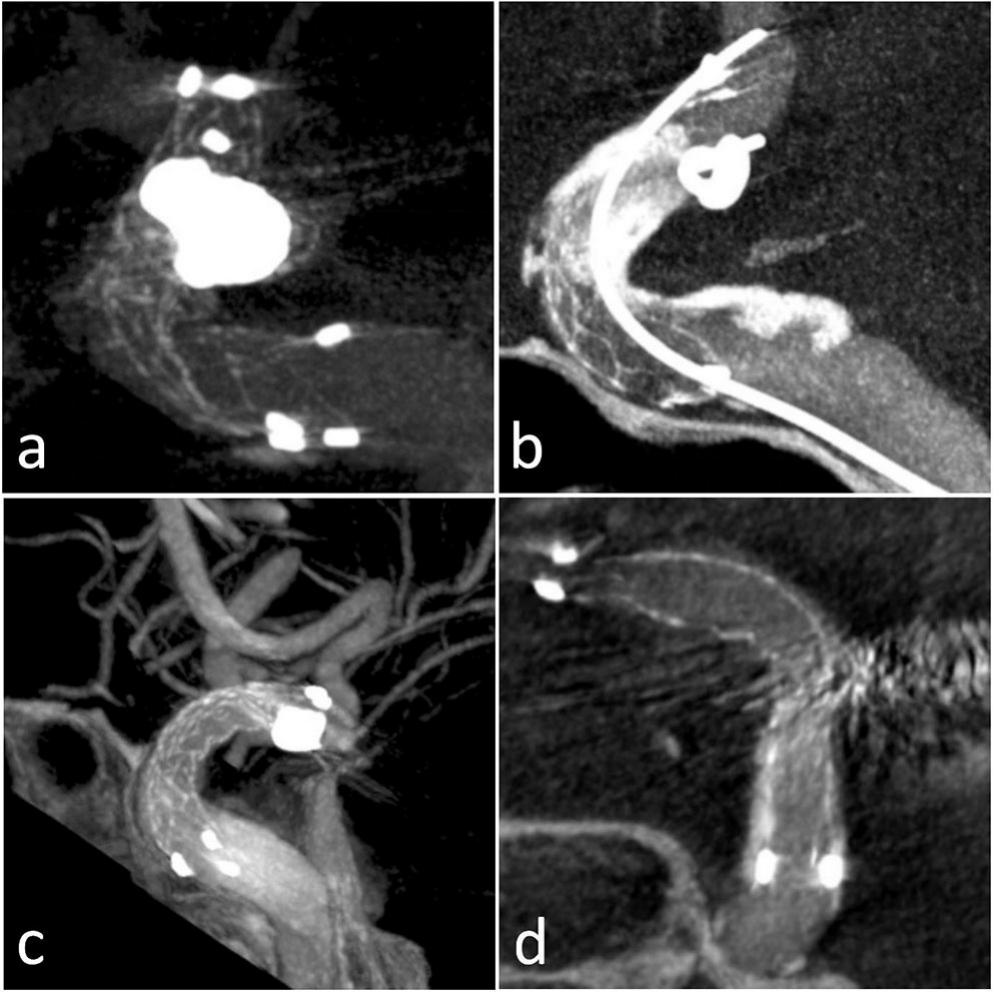
Four patients with ISA presentation in Xper CT: **(a)** Case 2 outer curve ISA; **(b)** Case 5 inner curve ISA; **(c)** Case 14 inner ISA; **(d)** Case 15 outer curve ISA.

Twenty-five patients with intracranial or cranial cervical junction aneurysms were included. Fourteen patients were male, with ages ranging from 48 to 78 years (mean ± SD, 65.08 ± 9.08 years). All aneurysms were unruptured except two. Treatments were performed successfully in all patients. Patient demographics and parent aneurysm artery features including location, diameter, radius, arc angle, stent details, follow-up information, and complications are shown in Table 1. Two aneurysms were located at the cervical segment of ICA, one at the petrous segment, seven at the cavernous segment, seven at the ophthalmic segment, seven at the posterior communicating segment, and one at the transverse segment of the vertebral artery. Among five patients with a stenosis of the parent artery (two mild, three severe), only one patient's stent was incompletely apposed. All were examined using Xper CT to confirm the stent wall apposition (Figure 1). Five patients had accompanying atherosclerotic stenosis. Stent kinking was observed in two aneurysms using Xper CT during the operation that were then corrected by massaging the stent via microcatheter or microwire (Figure 2). Mild crescent signs in four aneurysms were all implanted the 23-mm stent (Figure 3). The reason was that the larger angle of arc was subtended by the stent with the smaller curvature radius, which were determined according to a previous study (14).

**Table 1:** Summary of patient data and details of vessel anatomy.

**No**.	**Sex**	**Target lesion**	**stenosis**	**Stent length (mm)**	**Diameter (mm)**		**Angle**	**Radius(mm)**	**Stent markers**		**Stent apposition**		**Initial RROC**	**FU-RROC**	**Complication**	
					**Distal**	**Prox**.			**Prox**.	**Distal**	**Inner**	**Outer**			**Procedural**	**Post**
1	M	C7	-	23	3.26	4.7	61	4.63	A	A	C	C	I	I	-	-
2	F	C6	-	23	2.8	4.1	78	2.8	A	A	C	I	I	I	-	-
3	M	C4	-	30	2.89	3.22	87	3.39	A	A	C	C	I	I	-	-
4	M	C7	-	30	3.68	4.51	96	4.63	A	A	C	C	I	I	-	-
5	M	C7	Mild	23	2.88	4.04	139	2.96	A	A	I	C	I	I	Kinking	-
6	M	C4	Severe	30	3.31	3.67	71	3.35	A	A	C	C	I	I	-	-
7	F	C4	-	39	3.50	6.15	150	5.71	A	S	C	C	II	II	-	-
8	M	C6	-	23	3.53	4.83	36	3.51	A	A	C	C	I	N/A	-	-
9	F	C7	Severe	30	3.44	5.6	76	3.78	S	A	C	C	I	I	-	-
10	F	C6	Severe	23	3.74	3.96	70	3.85	A	S	C	C	I	I	-	-
11	F	C4	-	39	2.6	3.96	79	3.83	A	A	C	C	I	I	-	-
12	F	C6	-	23	3.35	4.78	56	4.3	S	A	C	C	I	I	-	-
13	M	C7	-	23	3.55	4.32	120	3.48	A	A	C	C	I	N/A	-	-
14	F	C7	-	23	3.71	4.45	101	3.8	A	A	I	C	I	I	-	-
15	F	C7	-	23	4.67	5.04	125	4.3	S	A	C	I	I	I	Kinking	-
16	F	C6	-	23	3.54	4.93	109	3.67	S	A	C	C	I	I	-	-
17	M	C6	-	23	3.66	4.63	100	3.84	S	A	C	C	I	I	-	-
18	M	C4	-	23	3.37	4.63	104	3.98	S	S	C	C	I	I	-	-
19	M	C2	-	39	5.12	5.69	150	5.83	S	S	C	C	I	I	-	-
20	M	C1	-	30	3.90	4.59	141	4.01	S	S	C	C	I	I	-	-
21	M	C1	-	30	4.42	4.66	97	4.65	S	S	C	C	I	I	-	-
22	F	V2	-	30	4.23	4.54	-	-	S	S	C	C	I	I	-	-
23	M	C4	-	30	3.56	3.98	-	-	S	S	C	C	I	I	-	-
24	M	C6	Mild	30	3.42	3.89	-	-	S	S	C	C	I	I	-	-
25	F	C4	-	16	3.76	4.03	-	-	S	S	C	C	I	I	-	-

*C1-C7 the seven segments of ICA according to Heller and Malek (14), RROC Raymond-Roy occlusion classification, A asymmetrical, S symmetrical, C complete, I incomplete, V2 the transverse segment of the vertebral artery, N/A non-applicable, FU follow up, Angle of arc subtended by stent and curvature radius were determined according to Roy and Milot (13)*.

Incomplete stent apposition occurred in four aneurysms. Possible related factors were compared (Table 2). There was significant difference in stent length between complete apposition group and incomplete apposition group (*P* = 0.001). A length of 23 mm appeared to be associated with ISA. No significant differences were found in terms of degree of stenosis, angle, radius, distal and proximal diameters, or diameter gradients of the parent artery.

**Table 2:** Comparison of complete apposition and incomplete apposition.

		**Complete apposition(*n* = 21)**	**Incomplete apposition(*n* = 4)**	***P*-value**
Stenosis		0.48 ± 1.08	0.25 ± 0.50	0.689
Stent length		27.95 ± 6.09	23 ± 0	0.001
Artery diameter	Distal	3.61 ± 0.52	3.52 ± 0.87	0.764
	Proximal	4.54 ± 0.69	4.41 ± 0.46	0.726
Artery angle		94.29 ± 32.72	110.75 ± 26.89	0.364
Artery radius		4.14 ± 0.74	3.47 ± 0.71	0.113
Diameter gradient		0.93 ± 0.66	0.89 ± 0.42	0.925

*Stenosis means the stenosis degree of parent artery: normal-0, mild-1, moderate-2, severe-3. The angle and radius of parent artery were the same data in Table 1. Diameter Gradient is the difference between the distal diameter and proximal diameter of parent artery*.

**Figure 4:**
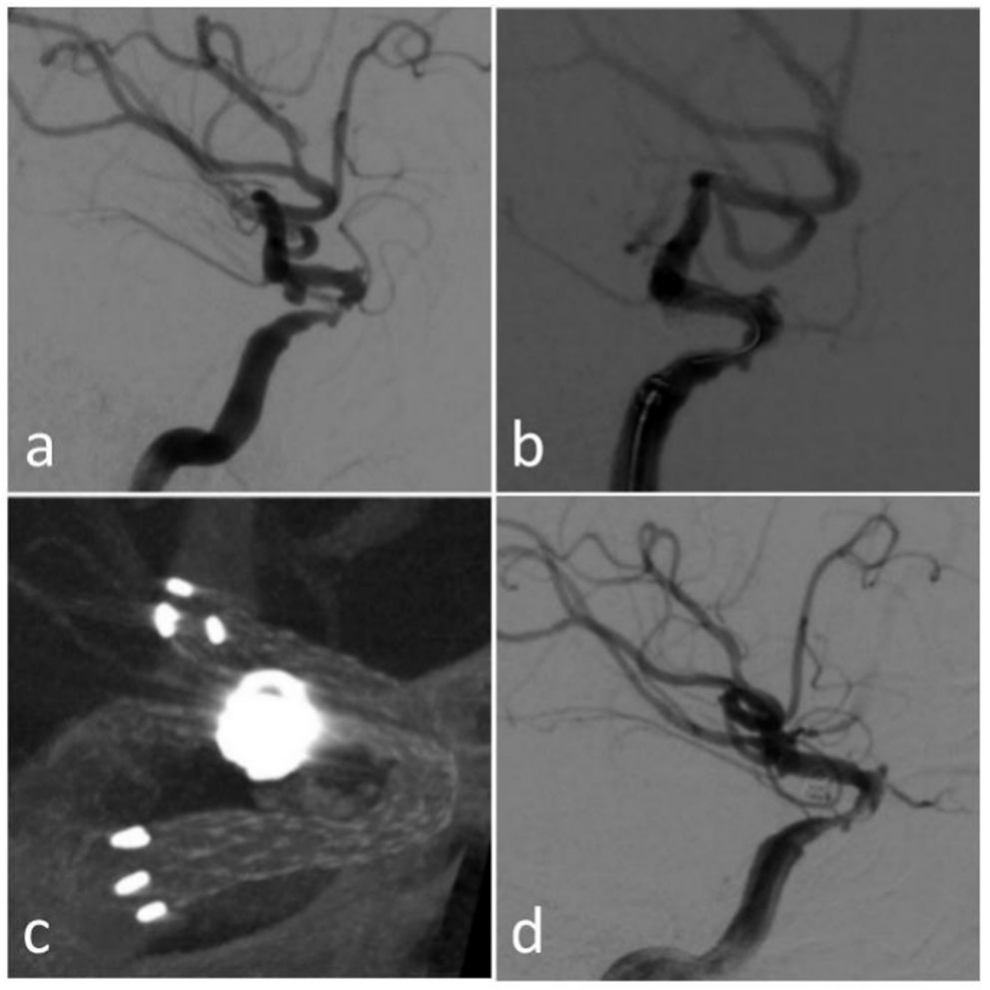
Case 9 **(a)** original DSA; **(b)** immediate angiography after the endovascular treatment; **(c)** Xper CT during the procedure; **(d)** follow-up angiography after 3 months.

**Figure 5:**
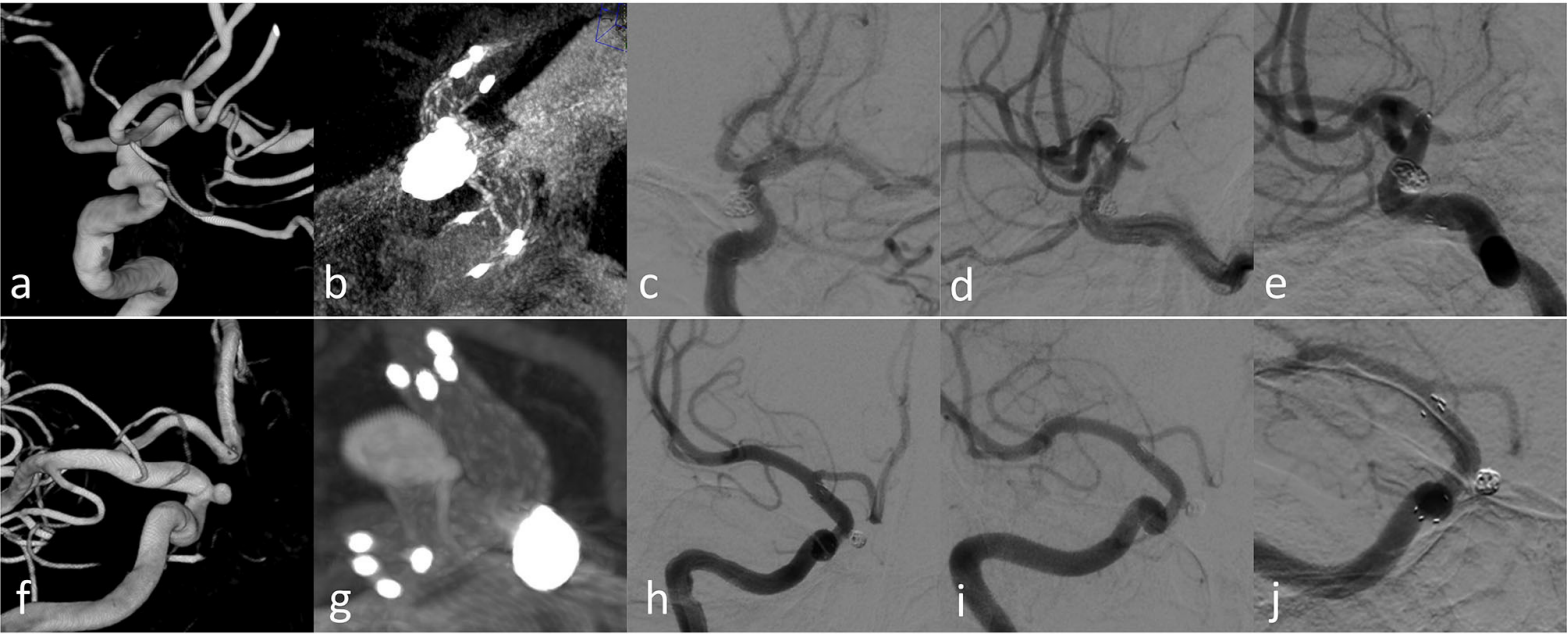
Case 3 **(a–e)** and Case 12 **(f–j)** with longer-term radiographic follow-up: **(a,f)** 3D images before treatment; **(b,g)** Xper CT immediately after SAC treatment; **(c,h)** immediate angiography after the endovascular treatment; **(d,i)** follow-up angiography after 3 months; **(f,j)** follow-up angiography after 12 months.

No patient underwent ischemic event after the operation. Two ruptured patients with acute treatment recovered well without developing further rupture or hemorrhage during hospitalization. Twenty-four aneurysms showed angiographic complete occlusion (RROC1) immediately after procedure, and one patient (Case 7) had residual neck filling (RROC 2) due to multiple nearby aneurysms on the same parent artery. Twenty-three patients were evaluated after 3–6 months by DSA. Among them, 22 showed RROC1 and Case 7 had residual neck filling with slight increase (RROC2). One re-stenosis was observed in the follow-up DSA with severe stenosis of the parent artery (Figure 4). Two patients (Case 3 and Case 12) were back after 3 and 12 months by DSA. Both showed RROC1 and had no stenosis on the parent arteries (Figure 5). No migration of the stents was observed during the deployment or follow-up.

## Discussion

Stent-assisted coiling has been demonstrated to have a higher rate of complete occlusion and a lower rate of recurrence in the long-term follow-up compared with conventional coiling especially for treatment of wide-necked aneurysms (15). However, it remains challenging because of potential stent-related embolic events of the parent artery (16). The common stents include Neuroform EZ, Enterprise, Lvis, Solitaire, Pipeline, and other flow diverters.

Diameters of wide-necked aneurysms located in the clinoid segment of ICA are usually about 5 mm or more (17), which the sizes of EP-VRD and Neuroform EZ cannot reach. Solitaire, a retrial stent with a large mesh, may lead to coil herniation and arterial occlusion if herniation loops are tangled inside the stent, giving rise to unsatisfactory complete occlusion rate on long-term follow-up (18). The rate of all thromboembolic events and the risk of in-stent stenosis were higher with the LVIS stent than with the Enterprise stent (16). A pipeline device and other flow diverters have been indicated for the endovascular treatment for large or giant wide-necked intracranial aneurysms in the petrous to the superior hypophyseal segments of ICA demonstrated by the Food and Drug Administration in 2011(19).

**Figure 6:**
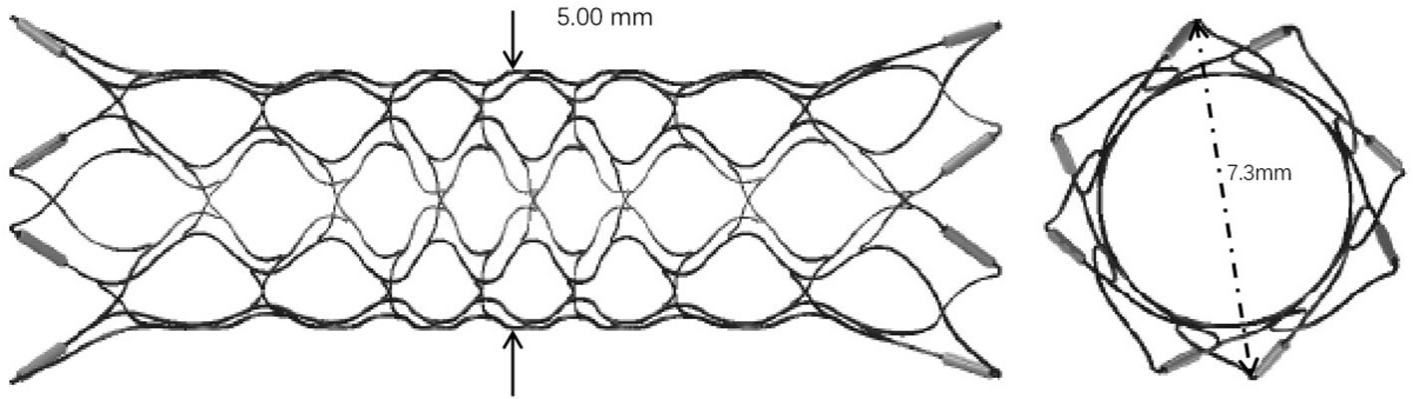
EP-VRD2 diameter extends to 5 mm, the diameter of the flare ends expands to 7.3 mm.

Enterprise VRD2 extended the body diameter of EP-VRD from 4.5 to 5 mm and enlarged the expanded diameter of the flare ends from 7 to 7.3 mm (Figure 6). Though the adaptation vessel diameters of both types remain the same, from 2.5 to 4.5 mm, the EP-VRD2 is supposed to perform better in larger vessels than EP-VRD (11). In our series, 21 of 25 aneurysms were located in the carotid siphon, the proximal diameter ranged from 3.22 to 5.60 mm, and the mean diameter was 4.53 mm, which was the most frequent location. Two stents were located in the cervical segment with the proximal diameter of the parent artery larger than 4.5 mm (one was 4.59 mm, the other 4.66 mm).

Enterprise, with a closed-cell design, has been shown to recapture and reposition including complete withdrawal and ovalization in curved vascular segments to keep coils staying within aneurysms, protecting the parent artery (20). However, the incomplete stent apposition (ISA) could also occur (20) associated with its closed-cell design and the angle of the vessel, which could be observed in the radiological images as well as clinical signs such as ischemic stroke or TIA, immediately (21) and on follow-up (9). Heller et al. detected ISA of the Enterprise on 3T-MRA with an unsatisfactory rate in 19/39 patients (49%) (14). EP-VRD2, a modified version, was designed to improve its apposition performance and reduce the incidence of ISA.

An *in vitro* study (11) found that EP-VRD2 achieved better vessel wall apposition than did the EP-VRD. In the present study, some ISAs (16%, 4/25) were observed on the parent artery in treatments with EP-VRD, two even using the pushing over outer curve technique during the deployment of the stent. However, none of the patients experienced cerebral ischemic events.

The angle and the radius of the parent artery have a significant influence on the wall apposition of the stent. The EP-VRD, in turn, exhibited significant kinking (consecutive flattening and narrowing of the stent lumen) when coming across an angle of 90° and more (14). In our cases, stent kinking occurred in two aneurysms during the procedure by Xper CT. Heller et al. demonstrated this phenomenon also *in vivo* (14) and found that this phenomenon was more likely to occur in larger vessels (4 mm vs. 3.7 mm), constituting curves with smaller radius (7.5 mm vs. 9.8 mm) and subtending large angles (140° vs. 95°) (9). The struts of EP-VRD2 were designed to allow the stent to elongate more on the outer curvature and to compress more on the inner curvature to increase its flexibility and its adherence to the vessel wall. We observed kinking of EP-VRD2 by Xper CT in two patients; one (case 5) had a small radius (2.96 mm), a larger angle (139°), and a larger difference between the radius of proximal (4.04 mm) and distal (2.88 mm) of the parent artery. This was eliminated by massaging the stent by a microcatheter. The other kinking was on the second curve of the parent vessel, which was also improved by massaging the stent by a “J-type” microwire. EP-VRD2, with a closed-cell stent, can also be repositioned and adjusted after the deployment, which cannot be achieved when using the open-cell stents as Neuroform EZ.

All stents were deployed by using the pushing over outer curve technique. Mild crescent signs (CS) were observed in four cases indicating ISA in Xper CT: Case 2 had the smallest radius of 2.8 mm but a small curve angle of 87°, Case 5 had a large angle of 139° and a smaller radius of 2.96 mm, and Cases 14 and 15 had a larger vessel diameter and a little smaller radius. All four cases used the stent length of 23 mm while the proximal markers were all located asymmetrically. The stent length influenced the stent apposition significantly especially for the length of 23 mm. Perhaps we should avoid the use of the 23-mm EP-VRD2. However, none of these four patients developed new neurological ischemic events, either immediately or on follow-up (3–6 months). More cases are needed to perform statistical analysis to validate this finding. We also observed that the stent lumen could be completely apposed to the vessel wall; even the proximal markers were located asymmetrically. This finding was similar to that of a previous study (12). In particular cases of EP-VRD, secondary migration was observed (22), while no migration was observed at deployment or on follow-up of EP-VRD2.

### Limitations

Our study has some limitations. First, our main limitation is the relatively small number of cases; however, it remains important to have a preliminary evaluation of this new stent in Chinese people. Second, angiographic evaluation of mid-term and long-term follow-up is needed to confirm its efficacy on recurrence rate and restenosis rate especially for those with stenosis. Third, Xper CT was not performed in the follow-up angiography especially for those four ISA patients and two stent-kinking patients during the procedural.

## Conclusion

EP-VRD2 stent may be a safe and effective device for embolization of intracranial wide-necked aneurysms with a good performance in large vessels that possess a relatively small-curved vessel radius. However, incomplete vessel wall apposition also occurred in the curves with the larger angle and smaller radius.

## Data Availability Statement

The original contributions presented in the study are included in the article/supplementary materials, further inquiries can be directed to the corresponding author/s.

## Ethics Statement

The studies involving human participants were reviewed and approved by Ethics committee of Zhejiang Hospital. The patients/participants provided their written informed consent to participate in this study.

## Author Contributions

LC and SW: planning, conduct, and reporting of the work described in the article. CZ, JW, JG, and YG: reporting of the work described in the article. All authors contributed to the article and approved the submitted version.

## Conflict of Interest

The authors declare that the research was conducted in the absence of any commercial or financial relationships that could be construed as a potential conflict of interest.
